# Cationic Polymer Micelles as Carriers of Bioactive Sesquiterpene Lactones from *Inula Helenium* L. for Effective Treatment of Bacterial Biofilms

**DOI:** 10.3390/pharmaceutics17060800

**Published:** 2025-06-19

**Authors:** Rumena Stancheva, Tsvetozara Damyanova, Tsvetelina Paunova-Krasteva, Ralitsa Veleva, Tanya Topouzova-Hristova, Viktoria Ivanova, Antoaneta Trendafilova, Ivaylo Dimitrov, Stanislav Rangelov, Emi Haladjova

**Affiliations:** 1 Institute of Polymers, Bulgarian Academy of Sciences, Akad. G. Bonchev St, Bl. 103A, 1113 Sofia, Bulgaria; rstancheva@polymer.bas.bg (R.S.); dimitrov@polymer.bas.bg (I.D.); rangelov@polymer.bas.bg (S.R.); 2 Centre of Competence “Sustainable Utilization of Bio-Resources and Waste of Medicinal and Aromatic Plants for Innovative Bioactive Products” (BIORESOURCES BG), 1000 Sofia, Bulgariatopouzova@biofac.uni-sofia.bg (T.T.-H.); viktoria.genova@orgchm.bas.bg (V.I.); antoaneta.trendafilova@orgchm.bas.bg (A.T.); 3 Stephan Angeloff Institute of Microbiology, Bulgarian Academy of Sciences, Akad. G. Bonchev St, Bl. 26, 1113 Sofia, Bulgaria; tsvetozaradamianova@gmail.com (T.D.); pauny@abv.bg (T.P.-K.); 4 Faculty of Biology, Sofia University St Kliment Ohridski, 8 Dragan Tsankov Blvd., 1164 Sofia, Bulgaria; 5 Institute of Organic Chemistry with Centre of Photochemistry, Bulgarian Academy of Sciences, Akad. G. Bonchev St, Bl. 9, 1113 Sofia, Bulgaria

**Keywords:** cationic polymer micelles, drug delivery, bacterial biofilms, phytochemicals, *Inula helenium* L.

## Abstract

**Objectives:** Nanosized polymeric micelles (PMs) with an average size of about 80 nm and moderately positive ζ potential, based on an amphiphilic poly(4-methyl-piperazin-1-yl)-propenone)-b-polylactide (PMPP-PLA) block copolymer, were prepared. They were used as platforms for the delivery of bioactive sesquiterpene lactones from *Inula helenium* L. root extract. **Methods:** The PMs were characterized with good encapsulation efficiency as a maximum value of 72% was reached at a polymer-to-extract mass ratio of 10:1. The loaded micelles exhibited good colloidal stability. An in vitro release was performed showing a burst release profile. The biocompatibility of the resulting PMs was confirmed by assessing their cytotoxic effect on human keratinocytes in vitro by colorimetric assay and flow cytometry. **Results**: The systems demonstrated the capability to reduce the biomass of pre-formed Gram-positive and Gram-negative bacterial biofilms. **Conclusions:** The obtained data clearly determine a trend for a strong combined effect between the PMs and the root extract, distinguishing them with an excellent anti-biofilm potential and prospects for future applications in medical practice.

## 1. Introduction

Fighting bacterial infections has been a problem for decades, and in recent years it has become even more serious due to the increasing antibiotic resistance of bacteria [1,2]. Bacterial infections are often caused by the formation of bacterial biofilm [3,4], characterized by a specific structure representing a sessile community of bacterial cells held together by an extracellular matrix [3,4,5]. Thus, the bacterial biofilm usually remains unaffected by conventional treatment with active substances. Therefore, the effective overcoming of the bacterial extracellular matrix barrier and the delivery of antibacterials present a significant challenge from a practical and scientific point of view.

Polymeric micelles (PMs) are well known as drug delivery carriers with applications in modern therapeutic preparations [6,7,8]. The advantages of PMs are due to their small size (typically from 10 to 100 nm) and their specific core–shell structure. They are composed of a hydrophobic core able to solubilize poorly soluble active substances, while the hydrophilic shell provides protection for the encapsulated substance, allowing its long-term circulation in the bloodstream [9,10]. In addition, the nanoscale size of PMs enables successful penetration through the cell membrane, making them effective in the transportation of cargo molecules such as drugs, nucleic acids, proteins, etc. [11]. PMs are characterized with good thermodynamic and kinetic stability, allowing improved pharmacokinetics and biodistribution high bioavailability, as well as controlled and targeted drug release [12,13]. In the past decade, PMs have been reported to find application as antibacterials [14,15,16]. Moreover, it has been found that PMs based on cationic polymers exhibit anti-biofilm properties themselves or could be loaded with antibiotics for combined treatment [17,18]. The main advantages of cationic PMs for the purposes of combating biofilms are related to their ability to destabilize and penetrate the bacterial membrane, leading to homogeneous diffusion of the active substance in the biofilm matrix [19]. Thus, a successful inhibition and destruction of bacterial biofilms could be achieved. A number of cationic PMs have been investigated in terms of their antibacterial and anti-biofilm properties against different pathogenic strains [20,21,22,23]. Their characteristic feature is that they do not induce bacterial resistance [24,25]. Nevertheless, increased cytotoxicity, associated with polycations, is usually the obstacle that limits their practical application [26,27].

Natural compounds of plant origin have demonstrated a wide range of activities, including anticancer, antimicrobial, antioxidant, anti-inflammatory, etc. [28,29,30]. They are relatively non-toxic, easily accessible, and can suppress resistance mechanisms, becoming a promising alternative to antibiotics. Moreover, in recent years natural compounds have increasingly been investigated as inhibitors of bacterial pathogenesis [19]. However, some issues, including low aqueous solubility, chemical instability, lipophilicity, size and structure, polar surface area, enzymatic reaction susceptibility, toxicity, etc. [31], may limit their individual usage as anti-biofilm agents. In this aspect, PMs could serve as vehicles, shielding the phytochemicals against oxidation in vitro and in vivo. Natural compounds such as resveratrol, curcumin, quercetin, etc., loaded into PMs show promising results in anticancer, antifungal, antiviral, and other treatments [32,33]. Therefore, in recent years, the usage of PMs as delivery vehicles for natural compounds has begun to develop.

**Figure 1:**
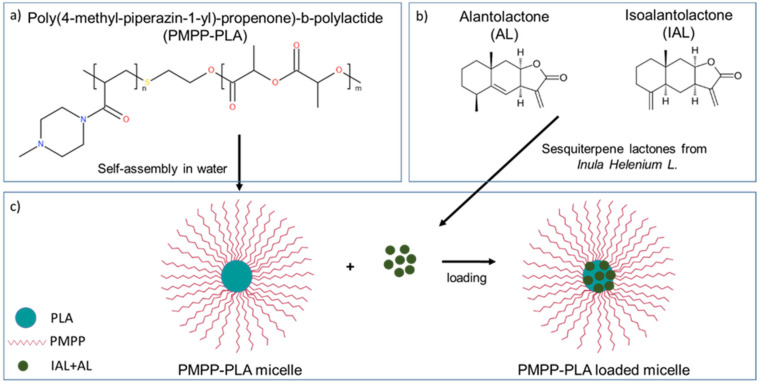
Chemical structures of PMPP-PLA block copolymer (**a**) and sesquiterpene lactones (AL and IAL) from *Inula helenium* L. (**b**) and a schematic illustration of PMPP-PLA-based polymer micelles’ structure and their loading with the root extract (**c**).

Poly(4-methyl-piperazin-1-yl)-propenone)-b-polylactide (PMPP-PLA) diblock copolymer (Figure 1a) has an amphiphilic character, forming narrowly distributed nanosized PMs in aqueous media [34]. These micelles have shown potential in gene delivery [34,35], demonstrating a prominent biocompatibility compared to other polycations. *Inula helenium* L. is a well-known medicinal plant used to treat respiratory and digestive diseases [36,37]. Its roots are rich in sesquiterpene lactones, primarily alantolactone (AL) and isoalantolactone (IAL) (see Figure 1b), and display a wide range of therapeutic properties, including antibacterial and antifungal [36,37]. Aiming at designing biocompatible polymeric anti-biofilm agents derived from plant extract that do not cause bacterial resistance, PMs based on the cationic PMPP-PLA copolymer were prepared and loaded with *Inula helenium* L. root extract. The polymeric systems were characterized in terms of their size and stability, ability to accommodate and release the plant extract, cytotoxic profile, as well as ability to inhibit the growth of pre-formed bacterial biofilms. A schematic illustration of this strategy is presented in Figure 1c.

## 2. Materials and Methods

### 2.1. Materials

#### 2.1.1. Inula helenium L. Root Extract

*Inula helenium* L. roots were purchased from a herbal drugstore www.bilki.bg (https://bilki.bg/oman-byal-koren.html, accessed on 20 September 2024). The roots (50 g) were finely ground and extracted with chloroform at room temperature. The crude extract (2 g) was obtained after filtration and concentration under reduced pressure. For HPLC analysis, 5.32 mg of the extract were dissolved in 2.5 mL CH_3_CN/CH_3_OH (4:1, *v*/*v*). The content of AL and IAL in the extract was found to be 235.9 and 211.5 mg.g^−1^ dry extract, respectively. A representative chromatogram is presented in Figure S1.

#### 2.1.2. PMPP-PLA Cationic Block Copolymer

The amphiphilic poly(1-(4-methylpiperazin-1-yl)-propenone)-b-poly(D,L-lactide) (PMPP-PLA) block copolymer (M_n_ = 4700 g·mol^−1^, Ɖ = 1.6) was synthesized according to a procedure described in detail elsewhere [34]. Briefly, 1-(4-methyl-piperazin-1-yl)-propenone (MPP) monomer was first polymerized under free-radical polymerization conditions in the presence of a 2-mercaptoethanol used as the chain-transfer agent. Next, the resulting poly(1-(4-methylpiperazin-1-yl)-propenone) (PMPP) homopolymer with a terminal hydroxyl group was used as a macroinitiator for the ring-opening polymerization of D,L-lactide. The molecular and physicochemical characteristics of the PMPP-PLA block copolymer are given in Table S1.

#### 2.1.3. Preparation of PMPP-PLA Micelles

PMPP-PLA micelles were prepared by the dropwise addition of block copolymer solution in tetrahydrofuran (Merck-Bulgaria, Sofia, Bulgaria) to an appropriate quantity of deionized water (ultra-pure water grade >18 MΩ) to obtain a final concentration of 0.5 mg·mL^−1^. The dispersion was subjected to extensive dialysis against water using SpectraPore 7 dialysis membranes (MWCO 8000, Repligen, Lund, Sweden) in order to remove the organic solvent.

#### 2.1.4. Loading of PMPP-PLA Micelles with *Inula helenium* Extract

The loading of PMPP-PLA micelles with *Inula helenium* extract was performed by adding an appropriate amount of the extract to the micellar dispersion (0.5 mg·mL^−1^), giving a polymer-to-extract mass ratio in the range of 1:1 to 25:1. The mixtures were sonicated for 1 h at 60 °C. Then, the water dispersions were filtered through sterile RC membrane filters with a pore size of 0.2 μm to collect the insoluble fraction of the root extract. Afterwards, the filters were rinsed with methanol (Merck-Bulgaria, Sofia, Bulgaria) to collect the non-loaded (free extract) fractions. The sesquiterpene lactones (AL and IAL) from the non-loaded *Inula helenium* fraction were quantified by high-performance liquid chromatography (HPLC) and the encapsulation efficiency (EE) was determined by the following equation:

$$EE\left(\%\right)=\frac{total\;amount\;sesquiterpene\;lactones-free\;amount\;sesquiterpene\;lactones}{total\;amount\;sesquiterpene\;lactones}\times 100$$

#### 2.1.5. Calculation of Solubility and Flory–Huggins Miscibility Parameters

The solubility parameter (δ) for the sesquiterpene lactones (AL and IAL) and core-forming hydrophobic polymer (PLA) was calculated by the group contribution method [38], using the following equation:

$$\delta =\rho \;\frac{\sum Fi}{M},$$

where ρ is the density of the sesquiterpene lactones (AL and IAL) or the polymer, M is the molar mass of the drug or polymer, and F is the molar attraction constant. For this study, δ was calculated using Hoy molar attraction constants [38].

The Flory–Huggins miscibility (χ) parameter was determined based on the Flory–Huggins theory [39] and calculated by the equation:

$$\chi =\frac{V_{d}}{RT}{\left(\delta_{d}-\delta_{p}\right)}^{2},$$

where V_d_ is the molar volume of the drug, δ_d_ and δ_p_ are the solubility parameters of the sesquiterpene lactone (AL or IAL) and core-forming hydrophobic polymer, respectively, R is the gas constant, and T is the temperature in Kelvin.

#### 2.1.6. In Vitro Release of Root Extract

The release of *Inula helenium* extract was performed in phosphate buffer (pH 7.4). The loaded PMPP-PLA micelles at polymer-to-plant extract mass ratio 10:1 (total volume of 3 mL) were placed in a dialysis membrane (SpectraPore 7, MWCO 50,000, Repligen, Lund, Sweden), and the membrane was immersed in 30 mL of the dissolution medium at 37 °C. Aliquots of samples were taken from the dissolution medium at specific time intervals, and that volume was replaced with fresh medium to maintain sink conditions. The amount of released sesquiterpene lactones (AL and IAL) from *Inula helenium* root extract was determined by HPLC.

### 2.2. Methods

#### 2.2.1. High-Performance Liquid Chromatography (HPLC)

HPLC analysis was performed on a Shimadzu Nexera-i LC-2040C 3D Plus liquid chromatograph equipped with a photodiode array detector (Shimadzu, Tokyo, Japan) and an analytical column Force C18 (150 × 4.6 mm, 3 µm) (Restek, Bellefonte, PA, USA).The temperature was maintained at 40 °C. The elution was carried out with a mixture of two solvents (phase A (H_2_O) and phase B (CH_3_CN)) [40] with slight modification: 0–30 min—55% B, 30–32 min—90% B, 32–37 min—90% B; 37–39 min—55% B; and 39–44 min—55% B. The flow rate was 0.8 mL.min^−1^ and the injection volume was 10 µL. The runs were monitored at 210 nm. The samples and mobile phases were filtered through a 0.22 µm membrane filter prior to analysis. Alantolactone (0.01–0.06 mg·mL^−1^, R^2^—0.9998) and isoalantolactone (0.01–0.06 mg·mL^−1^, R^2^—0.9997) at different concentrations were used as standards for the preparation of the calibration curves. All experiments were performed in triplicate.

#### 2.2.2. Dynamic and Electrophoretic Light Scattering (DLS and ELS)

DLS and ELS measurements were performed on a NanoBrook 90Plus PALS instrument (Brookhaven Instruments Corporation, Nashua, NH, USA) equipped with a 35 mW red diode laser (λ = 640 nm). DLS measurements were carried out at a scattering angle (θ) of 90° while the ζ potential measurements were carried out at a scattering angle (θ) of 15°. All measurements were performed at a temperature of 25 °C. The hydrodynamic diameter, D_h_, was calculated using the Stokes–Einstein equation. The ζ potentials were calculated from the obtained electrophoretic mobility by the Smoluchowski equation using the PALS method.

#### 2.2.3. Nanoparticle Tracking Analysis (NTA)

NTA measurements were performed on a NanoSight Pro analyzer (Malvern Panalytical, Worcestershire, United Kingdom) equipped with a high-sensitivity sCMOS camera. A 55 mW blue diode laser operated at 488 nm was used. The micellar dispersions were tenfold diluted before analysis. The samples were injected with sterile syringes through a NanoSight syringe pump (Malvern Panalytical, Worcestershire, UK), providing a continuous flow of particles (flow rate 3 μ·min^−1^) into the sample chamber. Measurements were performed at room temperature. The NanoSight Pro v1.0 software (Malvern Panalytical, Worcestershire, UK) was used for analyzing the data.

#### 2.2.4. Transmission Electron Microscopy (TEM)

The samples were examined using a TEM JEOL JEM-2100 (JEOL, Munich, Germany) electron microscope operating at 200 kV. They were prepared by depositing a drop of the micellar dispersion onto a carbon-coated grid followed by water evaporation at ambient temperature.

#### 2.2.5. In Vitro Cytotoxicity

HaCaT cell line was used to estimate the cytotoxicity of the empty polymeric micelles, loaded micelles (polymer-to-plant extract ratio 10:1), and pure *Inula helenium* root extract itself. Cells were incubated under standard conditions of 5% CO_2_, at 37 °C in DMEM supplemented with 10% FBS, and 1% antibiotic–antimycotic solution (penicillin 100 U·mL^−1^, streptomycin 100 μg·mL^−1^, and amphotericin B 0.25 μg·mL^−1^). Cells were seeded in 96-well plates with a concentration of 1 × 10^4^ cells per mL prior to experiments. After 24 h of incubation for adhesion and recovery, the cells were treated with concentrations of samples ranging from 25 to 125 µg·mL^−1^.

Cytotoxicity was assessed by two methods. For crystal violet assay, the cells were treated for 24 h, then washed with PBS and fixed with 4% buffered formaldehyde solution for 20 min. The samples were washed once again and stained with a 1% crystal violet solution for 20 min. They were air-dried after threefold washing, then the dye was dissolved with 10% acetic acid. The optical density of the samples was measured at 570 nm by an Epoch Microplate Spectrophotometer (BioTek, Winooski, VT, USA) with Gen5™ Data Analysis software, version 1.11.5. The results are presented as a percentage of cell viability compared to the control of untreated cells. The data was analyzed with OriginPro 9.0 and presented as a mean value ± SE. Statistical significance was calculated according to a one-way ANOVA at the 0.05 level (* *p* < 0.05). Flow cytometry was performed as another method to study cytotoxicity. Guava ViaCount reagent discerns between viable and non-viable cells and ensures viability data. The experiments were performed according to the manufacturer’s protocol. The cells were dissociated from the cell culture vessels with trypsin-EDTA solution and cell culture medium was added to a final concentration between 1 × 10^5^ and 1 × 10^7^ cells per mL. After adding the appropriate amount of reagent and incubation, the samples were measured on the Cytek^®^ Guava^®^ easyCyte™ flow cytometer (Luminex, Austin, TX, USA).

#### 2.2.6. Biofilm Experiments

The subject of this study includes two strains that cause various infections—*Staphylococcus aureus* ATCC 29213 and *Pseudomonas aeruginosa* ATCC 15692 [41]. Cultures which were 18 h old were used as the starting inoculum for studying biofilm dispersion. The strains were cultured in 96-well plates to form biofilms in the presence of minimal salt medium M63 and incubated for 24 h at 37 °C under static conditions, as previously described [18]. This was followed by triple washing to remove non-adherent cells and the loading of the treatment samples at a micellar concentration of 0.5 mg·mL^−1^. The applied treatment variants included empty micelles, loaded micelles (polymer-to-plant extract ratio 10:1), and pure *Inula helenium* L. root extract. The treatment was carried out for 24 h under incubation at 37 °C, followed by washing, crystal violet staining, solubilization, and measurement using an ELISA reader (LTEK INNO, Gyeonggi-do, Republic of Korea) at a wavelength of 595 nm.

## 3. Results

### 3.1. Preparation and Loading of PMPP-PLA PMs with Inula helenium L. Root Extract

**Figure 2:**
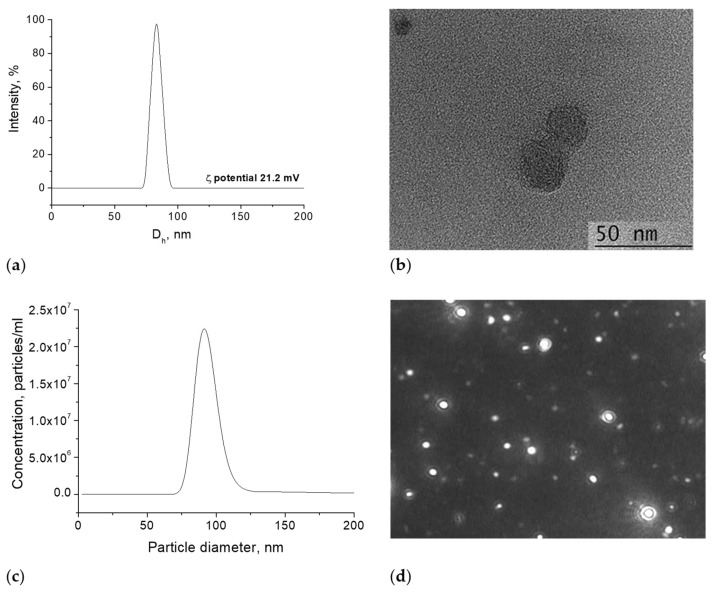
Size distribution curves obtained from DLS (**a**), NTA (**c**), TEM micrograph (**b**), and NTA image (**d**) of PMPP-PLA micelles prepared at concentration 0.5 mg.mL^−1^.

PMPP-PLA micelles were prepared by the dialysis method at a concentration of 0.5 mg.mL^−1^. The resulting particles were composed of a hydrophobic PLA core and hydrophilic cationic PMPP shell, as depicted in Figure 1c. DLS and ELS were used to determine the size and ζ potential of the PMPP-PLA PMs. It is known that the positively charged PMs with small size (<100 nm) can achieve deep infiltration into the biofilm matrix [10,14,15,17]. As evident from Figure 2a, the PMs are characterized with a narrow size distribution (PDI < 0.1) and a hydrodynamic diameter (D_h_) of 83 ± 6 nm. Due to the presence of tertiary amine groups in the PMPP structure, the micellar shell was positively charged, giving a ζ potential value of 21.2 mV. Therefore, the size and ζ potential of the resulting PMPP-PLA micelles meets the requirements for delivery systems used for bacterial biofilm destruction. Despite the weak polyelectrolyte properties of PMPP [35], the large surface area of the micellar shell provides sufficient surface charge density and the aqueous dispersion remains colloidally stable for more than 1 month, preserving the PMs’ size and ζ potential unchanged (see Figure S2). TEM was used for the visualization of particle morphology. A representative micrograph is shown in Figure 2b. As expected, the PMPP-PLA micelles were spherical in shape. According to the scale bar of the image the dimensions of the individual particles were about 40 nm, which is smaller than the size value determined by DLS. An explanation for this apparent disagreement between the two methods could be the state of the particles. In DLS, the particles were measured in their hydrated state, whereas during TEM sample preparation they were in a dried state, causing shrinkage and size reduction. NTA was performed to further characterize the PMPP-PLA PMs. This technique enables simultaneous particles size determination and their visualization in solution. The size distribution curve obtained from NTA is shown in Figure 2c. As evident, the curve maximum is at 87 nm, showing an excellent agreement with the DLS analysis. In Figure 2d, a picture taken during the particle tracking is shown, evidencing the spherical shape of PMPP-PLA PMs in aqueous medium together with their narrow size distribution.

The effective loading of poorly soluble active substances into PMs via hydrophobic interactions is predetermined by their thermodynamic compatibility. The determination of the Flory–Huggins miscibility parameter (χ) is a widely used method to consider the polymer–drug compatibility, thus it has become a criterion for loading driven by hydrophobic interactions. Therefore, the solubility (δ) and Flory–Huggins miscibility (χ) parameters of the hydrophobic components of the current systems were determined. As mentioned, the *Inula helenium* L. root extract is rich in sesquiterpene lactones (AL and IAL), which are responsible for its wide range of bioactive properties. The solubility parameters (δ) for the AL, IAL, and the hydrophobic core forming block (PLA) were calculated using the group contribution method. Then the δ values were used to determine the Flory–Huggins miscibility parameter (χ) for each couple. The data are summarized in Table 1. According to the Flory–Huggins theory, the lower the value of the χ parameter, the better the compatibility of the components. The χ value determined for PLA and the two sesquiterpene lactones were 1.22 and 1.28 for AL and IAL, respectively, revealing their good compatibility that is a prerequisite for effective loading.

**Figure 3:**
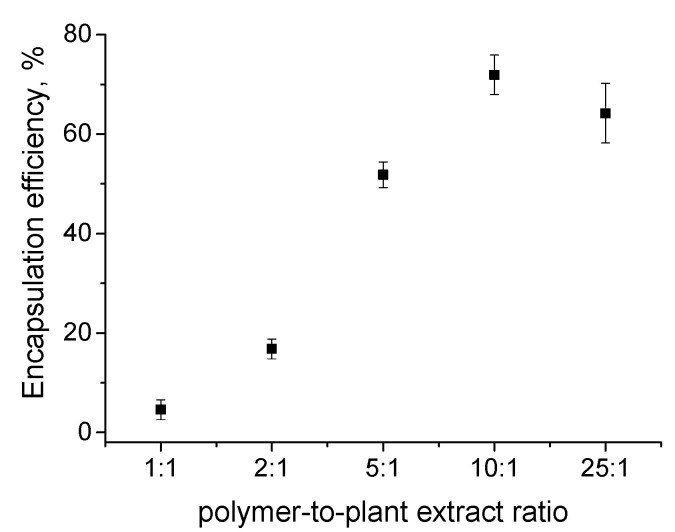
Variations of encapsulation efficiency as a function of polymer-to-plant extract mass ratio of PMPP-PLA micelles determined by HPLC. Each data point represents the arithmetic mean ± SD of three separate experiments.

The loading of *Inula helenium* in PMPP-PLA PMs was performed as the plant extract was added to the micellar aqueous dispersion, giving a polymer-to-plant extract mass ratio in the range of 1:1 to 25:1. The mixtures were sonicated for 60 min at 60 °C to ensure maximum solubilization then they were filtered to collect the insoluble plant extract fraction. The AL and IAL from the filter fractions were quantified by HPLC and used to determine the EE. As evident from Figure 3, the effect of the polymer-to-plant extract ratio on EE was noticeable. At lower ratios (1:1 and 2:1) a very low EE was achieved (below 20%). Increasing the polymer-to-plant extract ratio led to an increase in EE and an optimal value of 72% was reached at the 10:1 ratio. A further increase in the polymer-to-plant extract ratio (25:1) did not influence the value of EE. We could suggest that at lower ratios an optimum loading capacity of PMPP-PLA PMs is reached, limiting the adsorption of more Al and IAL molecules. Therefore, the drug loading content (DLC) was also calculated as the variations in this parameter as a function of the polymer-to-plant extract ratio are given in Figure S3. Indeed, in the range of ratios from 1:1 to 5:1 the DLC has the highest values—between 4 and 4.9%. At higher ratios (10:1 and 25:1) the DLC value decreases, giving rise to enhanced EE.

**Figure 4:**
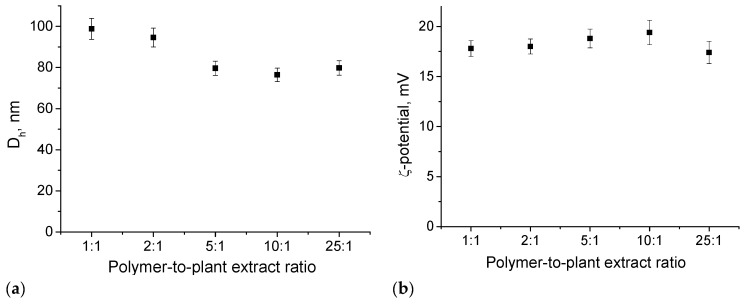
Variations of hydrodynamic diameter, D_h_, (**a**), and ζ potential (**b**) as a function of the polymer-to-drug mass ratio of PMPP-PLA micelles loaded with plant extract. Measurements were performed at 25 °C and pH 7. Each data point represents the arithmetic mean ± SD of three separate experiments.

DLS and ELS were used to follow the size and ζ potential of PMPP-PLA PMs after loading. The particle size variations with the polymer-to-plant extract ratio are given in Figure 4a. At low polymer-to-plant extract ratios (1:1 and 2:1) a slight increase in size (~10%) of loaded PMs was detected, while the D_h_ of particles at higher ratios (>5:1) was comparable to those of empty micelles. The increased PMs size could be attributed to the optimum DLC of the plant extract at lower ratios. A similar behavior has been observed for other polymeric micellar systems used as drug delivery vehicles [42]. The increased incorporation of AL and IAL molecules in the hydrophobic core of PMs at lower ratios leads to low colloidal stability of the systems. After 1 month, a precipitation was observed for particles loaded at 1:1 ratio (see Figure S4). For the rest of the loaded PMs no changes in the D_h_ were observed, showing their good colloidal stability. The ζ potential of micelles remains unchanged after loading (Figure 4b).

Based on the physicochemical characterization of the present micellar systems, the PMs loaded at a polymer-to-plant extract ratio of 10:1 were those with the most appropriate parameters (size, stability, EE, etc.). Therefore, for the subsequent experiments, PMs loaded at this ratio were used.

### 3.2. In Vitro Release of Inula helenium L. Root Extract

**Figure 5:**
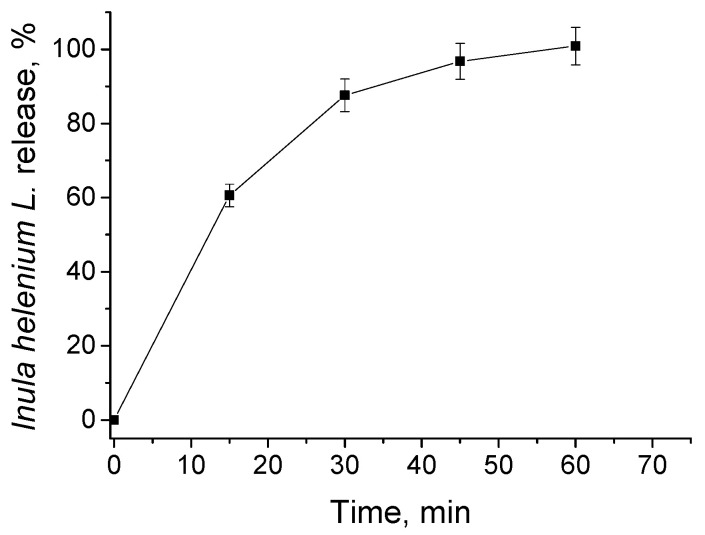
Release profile of *Inula helenium* L. root extract from PMPP-PLA micelles (loaded at a polymer-to-plant extract ratio 10:1) determined by HPLC analysis. The release was performed at 37 °C in phosphate buffer pH 7.4. Each data point in represents the arithmetic mean ± SD of three separate experiments.

The release of *Inula helenium* root extract from PMPP-PLA micelles was investigated at conditions resembling the extracellular fluid (phosphate buffer pH = 7.4, 37 °C). The amount of released plant extract was monitored by HPLC, quantifying the sesquiterpene lactones (AL and IAL). As evident from Figure 5, a burst release profile was observed and the plant extract was completely released within 60 min. Typically, the fast release from PMs is due to their disassembly [43,44]. However, under release conditions, the PMPP-PLA micelles are far above their CMC value (see Table S1). Therefore, release by diffusion of the plant extract through the micellar core could be suggested. The release data were then fitted into several kinetics models to investigate the mechanism of plant extract release. In Figure S5 plots giving Higuchi, Korsmeyer–Peppas, and zero-order kinetic models are shown. The best fit of the R^2^ value was found to be 0.964 for the Higuchi plot, proving our assumption of a diffusion-driven release mechanism. Here, it has to be mentioned that the length of the core-forming block is known to be a factor influencing the kinetic release of PMs-based delivery systems. In the case of currently investigated PMs, the PLA block of the copolymer used for their formation is short (only 11 units, see Table S1). This probably led to the formation of a porous core, facilitating the diffusion of AL and IAL molecules and accelerating their release, respectively.

### 3.3. Cytotoxicity Evaluation

**Figure 6:**
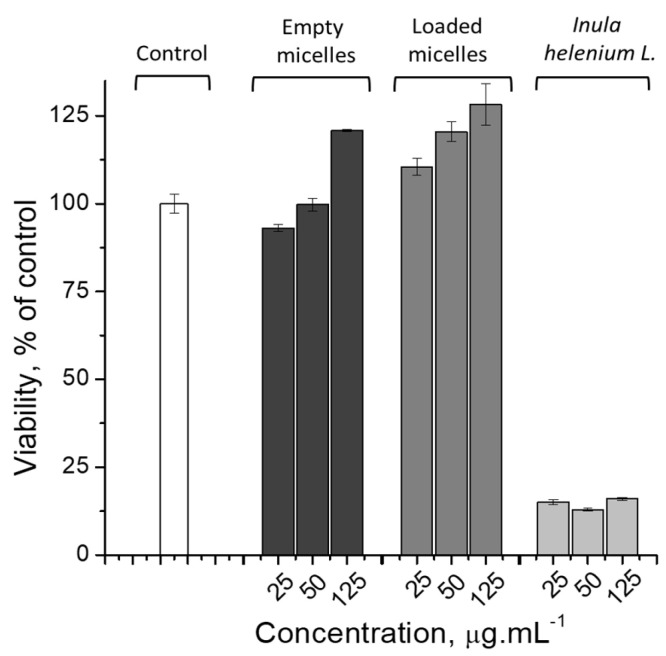
Cytotoxicity of empty PMPP-PLA micelles, the PMs loaded with *Inula helenium* root, and sole *Inula helenium* root extract on HaCaT cells. Crystal violet assay, performed after 24 h of treatment.

HaCaT cells are spontaneously immortalized human keratinocytes. These cells find usage in studying the effects of agents with antimicrobial activity and in studying substances with potential medical applications and beneficial effects on skin repair. Crystal violet assay was used to evaluate the cytotoxicity of the investigated systems, as this method is not affected by the constituents of the plant extract in the medium, unlike some enzyme-dependent cytotoxicity tests. According to the results presented in Figure 6, the cells treated with empty PMPP-PLA micelles and PMs loaded with *Inula helenium* L. root extract displayed a stronger signal than the control cells, which can be explained by the more intensive accumulation of actin microfilaments in response to the treatment. Crystal violet staining is sensitive to changes in the amount of DNA and actin filaments in the cytoplasm of cells, and the actin cytoskeleton is involved in the implementation of endocytosis, the likely route of entry of the studied particles. In contrast, the extract from *Inula helenium roots* tested alone showed high cytotoxicity towards actively proliferating keratinocytes, which confirms the well-known anti-proliferative effect of the sesquiterpene lactones presented in the roots of this plant. Treatment with *Inula helenium* L. root extract resulted in a viability of HaCaT cells below 20%. The low cytotoxicity of empty and loaded PMPP-PLA PMs was confirmed by flow cytometric examination of the tested concentrations (Figure S6). Regarding the *Inula helenium* L. extract, the ViaCount results show a clear dose-dependent cytotoxicity effect of the studied root extract on human keratinocytes.

### 3.4. Bacterial Biofilm Destruction

**Figure 7:**
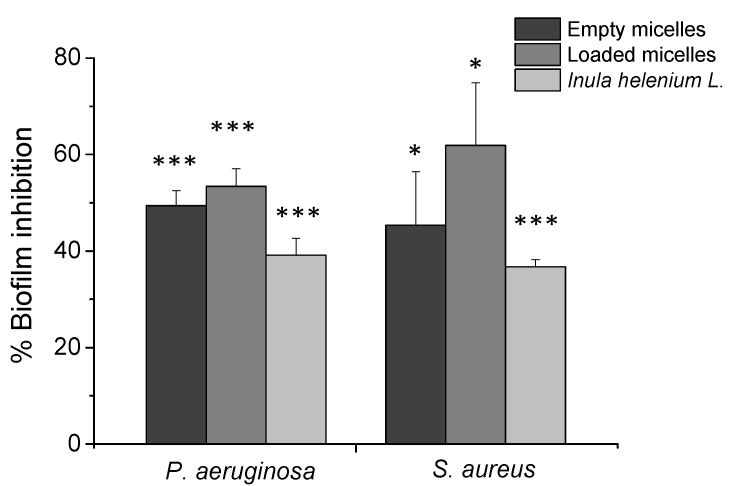
Anti-biofilm activity of empty PMPP-PLA PMs, PMs loaded with *Inula helenium* root extract at a polymer-to-plant extract ratio 10:1, and *Inula helenium* root extracts after 24 h treatment. Statistical analysis was performed using a one-way ANOVA test and a post hoc Tukey’s test (*p* < 0.001 ***) and (*p* < 0.05 *).

The effectiveness of empty and loaded PMPP-PLA PMs was investigated against biofilms of two strains, *P. aeruginosa* and *S. aureus*. Data analysis was performed after 24 h of treatment using crystal violet staining, and the concentration-dependent effects of the treated biofilms were calculated as a percentage relative to the untreated control samples for each strain (Figure 7). The results demonstrate the increased activity of PMs after loading against biofilms compared to empty micelles. The exfoliating effect of the loaded PMPP-PLA PMs exceeded 50% inhibited biofilm biomass for both treated strains, with higher values observed for *S. aureus* at 61.87%. The high efficiency values suggest that the loaded PMs probably facilitate targeted delivery of the encapsulated plant extract into the interior of pre-formed biofilms, regardless of the protective nature of the exopolysaccharide matrix. Empty PMPP-PLA micelles and *Inula helenium* root extract also exhibited effectiveness, with statistically significant inhibition rates of 49.4% and 39.1% for *P. aeruginosa*, and 45.3% and 36.7% for *S. aureus*, respectively.

## 4. Discussion and Conclusions

In the present study we propose an effective approach to enhance the therapeutic anti-biofilm efficacy of a plant extract, consisting of using of cationic block copolymer micelles as carriers of the extract. The block copolymer is composed of FDA-approved for clinical use PLA [45,46] as the hydrophobic block, and the weak polyelectrolyte PMPP, [47,48] with enhanced biocompatibility [34,35] compared to other polycations, as the water-soluble chain. The block copolymer spontaneously self-associated into sub-100 nm well-defined PMs (D_h_ = 83 ± 6 nm, ζ = 21.2 ± 2 mV, PDI < 0.1) that showed excellent tolerance towards human keratinocytes. *Inula helenium* L. is a widespread perennial plant widely used in traditional medicine. Its root extract shows anti-inflammatory, antioxidant, anthelmintic, anti-proliferative, antifungal, and cytotoxic activities and most of these biological properties are due to the presence of the sesquiterpene lactones (AL and IAL) [36,37]. Therefore, *Inula helenium* root extract possesses a prominent potential as an antibacterial, which is mainly limited by low solubility and high toxicity issues [49]. The low value of the Flory–Huggins parameter (Table 1) revealed good miscibility of the core-forming hydrophobic block and the sesquiterpene lactones from *Inula helenium.* L. root extract, allowing to reach an encapsulation efficiency as high as 72% at a 10:1 polymer-to-plant extract ratio. The loading of the plant extract in the PMs only barely influenced the particle characteristics (size and ζ potential, cf. Figure 1 and Figure 4), but it enhanced the viability of keratinocytes (Figure 6). Generally, the penetration of PMs through the biofilm matrix is governed by their size, surface charge, and hydrophobicity. PMs exhibit enhanced permeability as their nanoscale dimensions minimizes the diffusional barrier within the extracellular substance. The efficiency of PMs penetration, however, is also modulated by the structural characteristics of the biofilm matrix. In Gram-positive bacteria (e.g., *Staphylococcus aureus*, *Enterococcus facials*) the stability of the biofilm is critically dependent on the presence of extracellular proteins and extracellular DNA. The matrix architecture is relatively less compact compared to Gram-negative bacteria, despite the presence of a thicker exopolysaccharide layer. Key polysaccharide components include poly-β-1,6-N-acetylglucosamine and teichoic acids, which contribute to the overall negative charge of the biofilm matrix. In contrast, Gram-negative bacterial biofilms such as Pseudomonas aeruginosa are enriched in exopolysaccharides (alginate, Psl, and Pel). These are essential for bacterial adhesion and contribute to the highly organized matrix architecture, including the formation of fluid-filled channels and micro-colonies that increase matrix porosity. The negative surface charge in these biofilms is predominantly attributed to alginate and lipopolysaccharides [50,51,52,53]. Following matrix penetration and interaction with bacterial membranes, PMs disrupt membrane integrity, leading to the leakage of intracellular contents. Their insertion into the phospholipid bilayer and interactions with cellular components such as DNA, enzymes, and ribosomes result in the impairment of key metabolic processes, the induction of oxidative stress, electrolyte imbalance, enzyme inhibition, and ultimately a bactericidal effect [19]. Therefore, the small size and the positive ζ potential of the loaded PMPP-PLA micelles are beneficial for penetrating the exopolysaccharide barrier of mature biofilms disrupting essential cellular processes [53]. Furthermore, the high encapsulation efficiency of the PMs and, particularly, the burst release of the root extract from the PMs additionally strengthened the anti-biofilm effect for both strains studied (*P. aeruginosa* and *S. aureus*). Despite the fact that *Inula helenium* root extract possesses its own anti-biofilm activity, it is also characterized by prominent cytotoxicity towards keratinocytes, which disappeared upon loading in the PMs (Figure 6). Our previous studies have revealed the effectiveness of cationic PMs—empty or loaded with antibiotics—against biofilms of both Gram-positive and Gram-negative bacteria [18,19]. In another study using methanol extracts from *Inula salicina*, we demonstrated significant anti-biofilm activity against *S. aureus* [54]. With the investigated PMPP-PLA PMs loaded with the plant extract, strengthening of the exfoliating effect for the Gram-positive strain was observed. *S. aureus* is a well-known causative agent for various infections, including infectious endocarditis, osteomyelitis, meningitis, arthritis, pneumonia, urinary tract infections, and skin abscesses such as folliculitis and impetigo [55,56,57,58]. Its development is often associated with nosocomial and postoperative infections, frequently linked to the placement of implants [59,60,61]. *S. aureus* is among the most difficult bacteria to treat due to its high drug tolerance [62]. The expression of various virulence factors, adhesion, invasion, toxin synthesis, biofilm formation, and the ability to evade the cellular immune response enable this pathogen to multiply uncontrollably, leading to severe and often untreatable infections. At the same time, recent data indicate that the incidence of biofilm-related infections is rapidly increasing, leading to chronic conditions and significant challenges in treatment.

Our study offers an innovative inhibition process suitable for the dispersion of established biofilms. This approach differs from our previous studies, where we reported inhibition during the biofilm formation stage [54,63,64,65]. The findings are particularly important as a large percentage of biofilm-associated infections occur at a chronic stage where bacterial development has already progressed into a biofilm. Additionally, the applied model is promising as the impact of the inhibitory effect, produced by the loaded PMs, is greater than the individual effects of the two entities. The obtained data clearly indicate a trend of excellent dispersion potential against pre-formed biofilms and offer a broad scope for their application.

## Figures and Tables

**Table 1:** Solubility (δ) and Flory–Huggins miscibility (χ) parameters calculated for the hydrophobic core forming polymer (PLA) and sesquiterpene lactones (AL and IAL) from the *Inula helenium* root extract.

Polymer/Extract	δ [(MPa)^1/2^] *	χ_AL_	χ_IAL_
PLA	21.33	1.22	1.28
AL	17.6	-	-
IAL	17.5	-	-

* δ was calculated using Hoy molar attraction constants.

## Data Availability

Data are contained within this article.

## Supplementary Materials

The following supporting information can be downloaded at: https://www.mdpi.com/article/10.3390/pharmaceutics17060800/s1, Table S1: Degree of polymerization of hydrophilic and hydrophobic blocks, hydrophilic-lipophilic balance, and critical micellization concentration of PMPP-PLA block copolymer; Figure S1: HPLC chromatograms of a standard mixture of isoalantolactone and alantolactone, *I. helenium* root extract and *I. helenium* loaded in PMPP-PLA PMs; Figure S2: Size distribution curve obtained from DLS of PMPP-PLA micelles after 1 month storage; Figure S3: Variations in drug loading content as a function of polymer-to-plant extract mass ratio of PMPP-PLA micelles determined by HPLC; Figure S4: Variations in the hydrodynamic diameter with the polymer-to-drug mass ratio of PMPP-PLA micelles loaded with plant extract after storage for 1 month; Figure S5: Release kinetics of *Inula helenium* L. root extract from PMPP-PLA micelles fitted into kinetics models as follow: (a) zero order; (b) Higuchi model; (c) Korsmeyer―Peppas model; Figure S6: Cytotoxicity of empty PMPP-PLA micelles, PMs loaded with *Inula helenium* root extract, and *Inula helenium* extracts on HaCaT cells. Flow cytometry with Guava ViaCount reagent staining, performed after 24 h of treatment.
