# Supplementary material for: Cationic Polymer Micelles as Carriers of Bioactive Sesquiterpene Lactones from Inula Helenium L. for Effective Treatment of Bacterial Biofilms
Source: Pharmaceutics. 2025 Jun 19;17(6):800. doi: 10.3390/pharmaceutics17060800 (PMC12197024; doi:10.3390/pharmaceutics17060800)
Supplement: Supplementary file 1 [file pharmaceutics-17-00800-s001.zip › pharmaceutics-3692175-supplementary.pdf]

SUPPLEMENTARY MATERIALS

Cationic Polymer Micelles as Carriers of Bioactive Sesquiterpene Lactones from *Inula Helenium* L. for Effective Treatment of Bacterial Biofilms

R. Stancheva[1,5], Ts. Damyanova[2], Ts. Paunova-Krasteva[2], R. Veleva[3], T. Topouzova-Hristova[3,5], V. Ivanova[4,5], A. Trendafilova[4,5], I. Dimitrov[1], S. Rangelov[1,5], E. Haladjova[1,5]

[1] *Institute of Polymers, Bulgarian Academy of Sciences, Akad. G. Bonchev St, Bl. 103A, 1113 Sofia, Bulgaria*

[2] *Stephan Angeloff Institute of Microbiology, Bulgarian Academy of Sciences, Akad. G. Bonchev St, Bl. 26, 1113 Sofia, Bulgaria*

[3] *Faculty of Biology, Sofia University St Kliment Ohridski, 8 Dragan Tsankov Blvd., 1164 Sofia, Bulgaria*

[4] *Institute of Organic Chemistry with Centre of Phytochemistry, Bulgarian Academy of Sciences, Akad. G. Bonchev St, Bl. 9, 1113 Sofia, Bulgaria*

[5] *Centre of Competence "Sustainable Utilization of Bio-resources and Waste of Medicinal and Aromatic Plants for Innovative Bioactive Products" (BIORESOURCES BG), 1000 Sofia, Bulgaria*

Table S1. Degree of polymerization of hydrophilic (n) and hydrophobic (m) blocks, hydrophilic-lipophilic balance (HLB), and critical micellization concentration (CMC) of PMPP-PLA block copolymer. Data taken from Ref. 1.

**Copolymer**	**n**	**m**	**PMPP content, mol %**	**HLB**	**CMC, mg/ml**
**PMPP-PLA**	20	11	65	13.2	0.0172

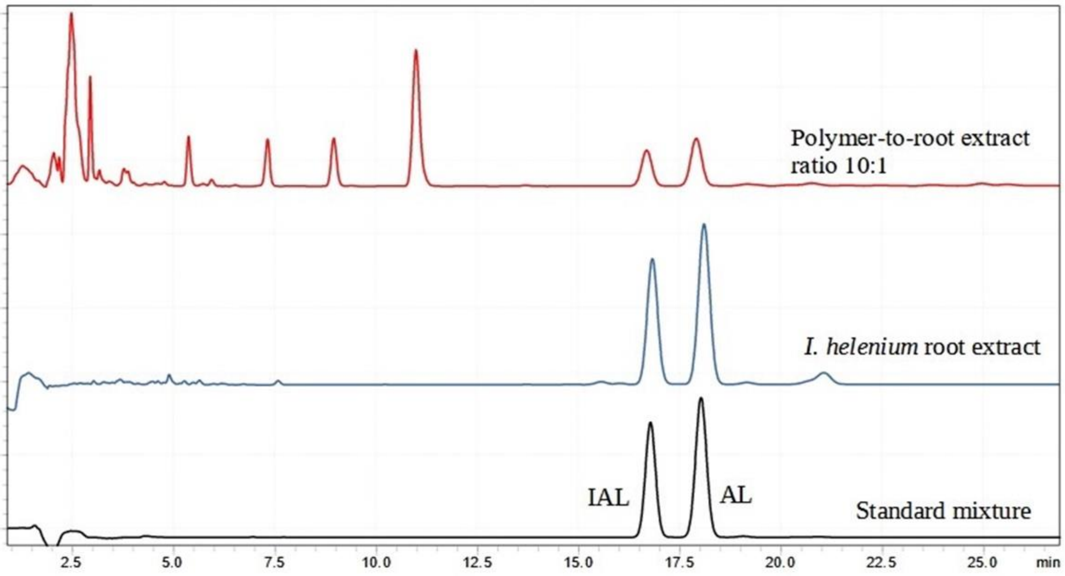

Figure S1. HPLC chromatograms of a standard mixture of isoalantolactone and alantolactone, *I. helenium* root extract and *I. helenium* loaded in PMPP-PLA PMs (polymer-to-root extract ratio 10:1) at 210 nm.

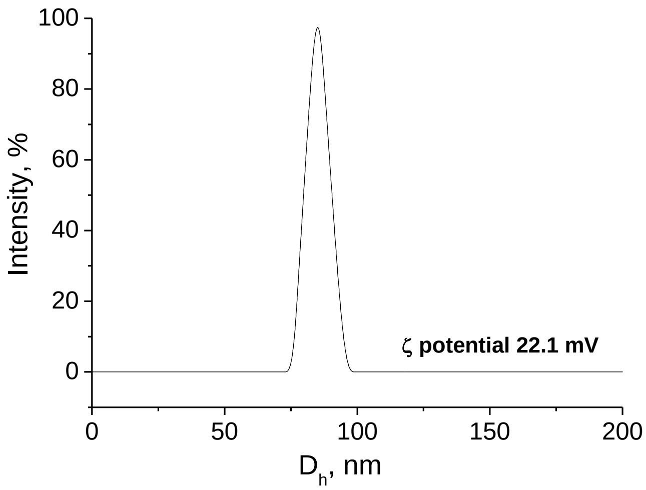

Figure S2. Size distribution curve obtained from DLS of PMPP-PLA micelles prepared at concentration 0.5 mg.mL^{-1} after 1 month storage.

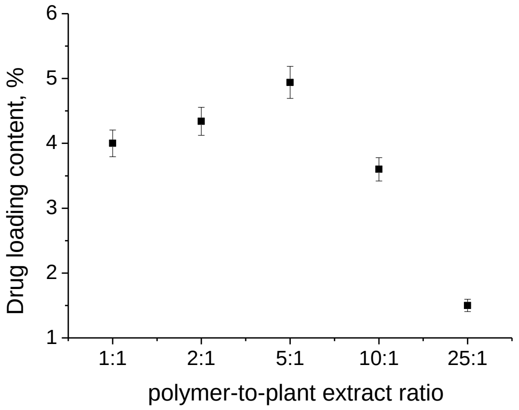

Figure S3. Variations of drug loading content as a function of polymer-to-plant extract mass ratio of PMPP-PLA micelles determined by HPLC. Each data point represents the arithmetic mean ± SD of three separate experiments.

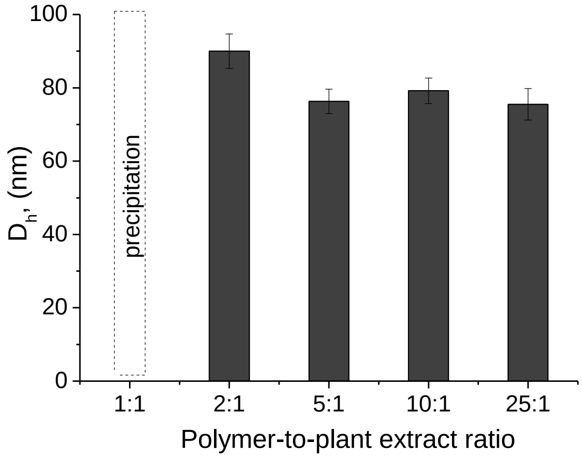

Figure S4. Variations of the hydrodynamic diameter, D_h, with the polymer-to-plant extract mass ratio of PMPP-PLA micelles loaded with plant extract after storage for 1 month. Measurements were done at 25 °C at pH 7. Each data point in represents the arithmetic mean ± SD of three separate experiments run in triplicate.

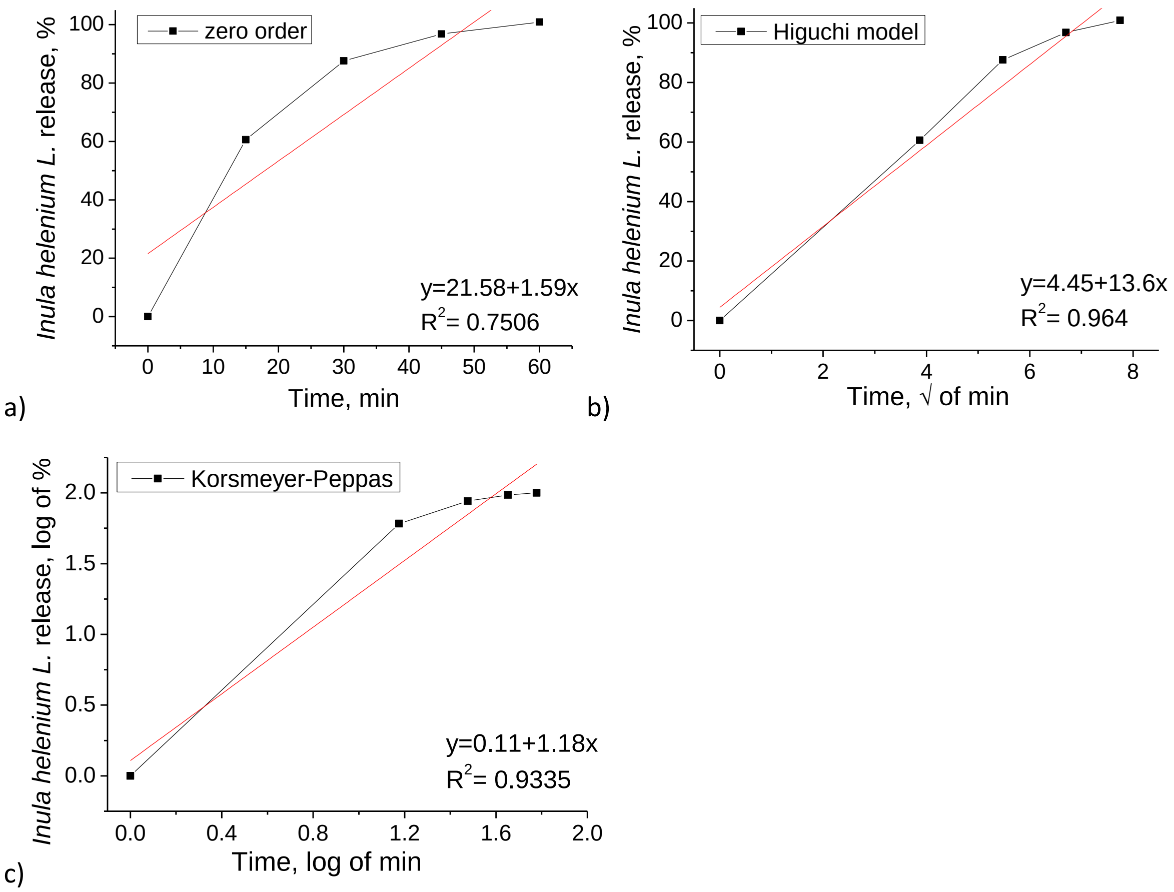

Figure S5. Release kinetics of *Inula helenium L.* root extract from PMPP-PLA micelles fitted into kinetics models as follow: a) zero order; b) Higuchi model; c) Korsmeyer-Peppas model.

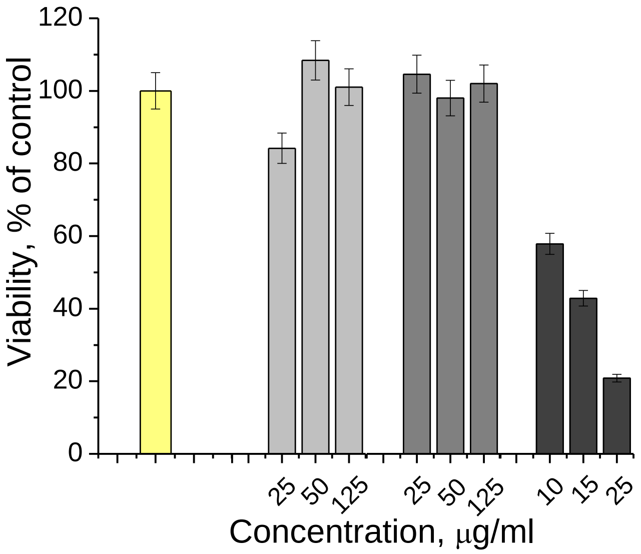

Figure S6. Cytotoxicity of empty PMPP-PLA micelles, PMs loaded with *Inula helenium* root extract and *Inula helenium* extract on HaCaT cells. Flow cytometry with Guava ViaCount reagent staining, performed after 24h of treatment.

References

1. Haladjova, E.; Dimitrov, I.; Davydova, N.; Todorova, J.; Ugrinova, I.; Forys, A.; Trzebicka, B.; Rangelov, S. Cationic (Co)polymers Based on N-Substituted Polyacrylamides as Carriers of Bio-macromolecules: Polyplexes, Micelleplexes, and Spherical Nucleic Acidlike Structures. Biomacromolecules 2021, 22, 971.
